# A study on the optimal design of isothermal experiments in predictive microbiology

**DOI:** 10.1038/s41598-025-15810-2

**Published:** 2025-08-20

**Authors:** Alba Muñoz del Río, Víctor Casero-Alonso, Mariano Amo-Salas

**Affiliations:** Department of Mathematics, Institute of Mathematics Applied to Science and Engineering, University of Castilla-La Mancha, Ciudad Real, Spain

**Keywords:** Parameter estimation, *D*- and *c*-optimality, Efficient designs, Final experimental time, Baranyi and Ratkowsky square-root model, Microbiology, Statistics

## Abstract

This study addresses from the Optimal Experimental Design perspective the use of the isothermal experimentation procedure to precisely estimate the parameters defining models used in predictive microbiology. Starting from a case study set out in the literature, and taking the Baranyi model as the primary model, and the Ratkowsky square-root model as the secondary, *D*- and *c*-optimal designs are provided for isothermal experiments, taking the temperature both as a value fixed by the experimenter and as a variable to be designed. The designs calculated show that those commonly used in practice are not efficient enough to estimate the parameters of the secondary model, leading to greater uncertainty in the predictions made via these models. Finally, an analysis is carried out to determine the effect on the efficiency of the possible reduction in the final experimental time.

## Introduction

In the field of food safety, predictive microbiology has become an essential tool^1^, as it is responsible for developing the models which allow the behaviour of the microorganisms in food to be described. These models can be divided into two main categories, primary and secondary models^2^. The primary models describe the growth or inactivation of the microorganisms in static or constant environmental conditions, and therefore these models are expressed only as functions of time. In the secondary models, the kinetic parameters of the primary model are expressed as functions of environmental factors, such as temperature, pH or water activity. Dolan and Mishra^3^ provides a comparison of the two types of model, with regard to the methodology for estimating the parameters, showing the main advantages and drawbacks of each one.

Experimentation is key to the construction of these models, since the quality of experiments is directly related to the precision with which model parameters are estimated. This is very important, as uncertainty in the estimations is reflected in the predictions made with these models^4^. Faced with this problem, many studies have considered using techniques of Optimal Experimental Design (OED). OED is a statistical methodology aimed at selecting the most informative experimental conditions according to specific optimality criteria. It allows experimenters to obtain parameter estimates that are unbiased and have minimum variance, using the fewest possible observations. In practical terms, this leads to reduced experimental costs and improved efficiency. In this context, many studies have considered using OED techniques to determine the best experimental points to obtain the maximum possible information, and thus more accurate estimations. The work of Versyck et al.^5^, pioneers in this area, applies OED in an Arrhenius-type secondary inactivation model. In the literature is widely used the combination of the Baranyi model as the primary model, and the Ratkowsky square-root model as the secondary, as it is the model type that has been most thoroughly analysed from the OED perspective, in both the isothermal and non-isothermal cases^6–10^. Nevertheless, there are also studies applying OED to other models, such as the modified Gompertz model^11^ or the Bigelow, Mafart and Peleg models^12^.

When carrying out these experiments, there are two different possibilities: isothermal or non-isothermal experiments. Although the design of the experiments has been studied for both possibilities, various authors indicate the former are more appropriate for estimating the kinetic parameters of the model, due to the constant conditions throughout the experiments^13,14^. In addition, they are less expensive and easier to perform and analyse. In contrast, non-isothermal experiments provide a more realistic view of microbial behaviour. It is important to note that, in both types of experiments, the observations are obtained from different units of the food product under study. This ensures the use of genuine replicates, meaning that each measurement is taken independently by repeating the same experiment under identical conditions, thus avoiding correlation between observations. The usual procedure found in the literature for modelling microbial behaviour is to carry out isothermal experiments for different temperatures and estimate the primary model parameters for each temperature. From the estimates of the kinetic parameter of the model for the different temperatures, the parameters of the secondary model are estimated. Once all the parameters have been fitted, the model is validated using non-isothermal experiments^15–17^. However, this procedure involves a significant problem from a statistical point of view, since it assumes the estimates of the kinetic parameter as observed values in order to perform the subsequent fitting of the parameters of the secondary model, ignoring the uncertainty of these estimates. It is clear that if, for each temperature, the estimates of the kinetic parameter of two experiments are equal, they will lead to the same estimates of the secondary model parameters regardless of the uncertainty in the first. However, if this inherent variability is taken into account, it is assumed that the lower the uncertainty in the estimates of the kinetic parameter, the lower the uncertainty in the estimates of the parameters of the secondary model, and the greater the confidence in the predictions made by the model.

The aim of this study is to analyse, from the perspective of OED, the different scenarios that can be considered when planning isothermal experiments, in order to accurately estimate the parameters of the primary and secondary models. In this study, it is considered the primary Baranyi model and as a secondary model the Ratkowsky square-root model, because they are the most widely used in the literature. These models are used with the data obtained in the growth experiments of *Pseudomonas spp.* on button mushrooms (*Agaricus bisporus*), described by Tarlak et al.^16^. This study shows both the optimal designs for each of these scenarios and the comparison between them, allowing the loss of efficiency that occurs in each case, and thus the robustness of the designs obtained, to be determined.

## Methods

### Mathematical models

**Fig. 1:**
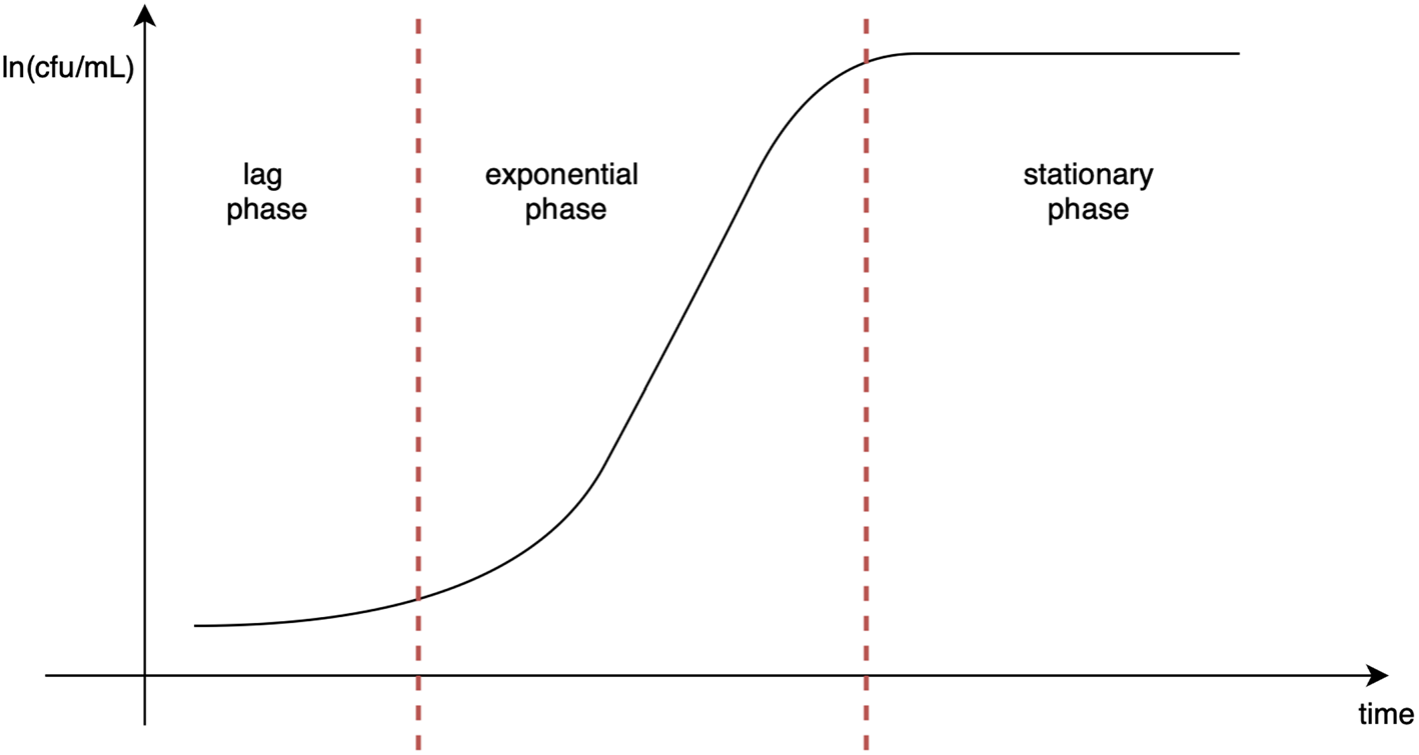
Microbial growth curve as described by the Baranyi model (1).

Among the models considered in the predictive microbiology literature, the Baranyi model stands out, due to its specificity and the physical meaning of its parameters. The explicit expression of this primary model^18^ is as follows:

1 $$\begin{aligned} y(t) = y_0 + \mu _{max}F(t) - \ln \left( 1 + \frac{e^{\mu _{max}F(t)} - 1}{e^{(y_{max} - y_0)}}\right) , \end{aligned}$$

with

$$\begin{aligned} F(t)= t + \frac{1}{\mu _{max}}\ln \left( e^{-\mu _{max} t} + e^{-\mu _{max}\lambda } - e^{-\mu _{max} t - \mu _{max}\lambda }\right) , \end{aligned}$$

where $y(t)$ is the natural logarithm of cell concentration (CFU/mL) at time $t$ (hours); $y_0$ and $y_{max}$ are the natural logarithms of initial and asymptotic concentrations, respectively; $\mu _{max}$ is the maximum growth rate and $\lambda$ is the lag phase duration (h). The function $F(t)$ is used to incorporate the adjustment for the lag phase in microbial growth. It ensures a smooth transition from the lag phase to the exponential growth phase, allowing the model to better represent real growth curves, especially at early time points. A graphical representation of the typical microbial growth curve is provided in Fig. 1, where the lag, exponential, and stationary phases are indicated to help visualize the dynamics captured by the Baranyi model. The Baranyi model can also be formulated in a differential form, which is commonly used in non-isothermal studies due to its flexibility for dynamically changing conditions.

With the aim of considering the effect of the temperature on the experiments, the Ratkowsky square-root model^19^ is taken as the secondary model. This describes the influence of suboptimal growth temperatures on the maximum specific growth rate of microorganisms ($\mu _{max}$). This model can be expressed as:

2 $$\begin{aligned} \sqrt{\mu _{max}(T(t))}=b(T(t)-T_{min}), \end{aligned}$$

where *T* is the temperature (°C), *b* is a regression coefficient and $T_{min}$ is the theoretical minimum growth temperature. Note that the Ratkowsky model is generally valid for suboptimal temperature ranges. Although more complex models, such as the extended Ratkowsky model^20^, may better describe microbial behaviour near maximum growth temperatures, the Ratkowsky model remains a standard choice for many applications.

### Optimal experimental design methodology

Starting from the premise that the total number of possible observations in an experiment is *N*, *exact design* of size *N* means a sequence of *N* points in $\mathscr {X}$, where $\mathscr {X}$ represents the set of observable points, also known as the design space, on which the controllable variables are evaluated. This design is denoted $x_1, \ldots , x_N$, where some of these points may coincide. If $N_x$ is the number of observations to be made at point *x*, we define it as the number of genuine replicates at that point, meaning independent repetitions of the experiment under identical conditions at *x*. The discrete measure associated with that design is expressed as follows:

$$\begin{aligned}\xi (x) = \frac{N_x}{N}, \quad x \in \mathscr {X}.\end{aligned}$$

This gives the definition of *approximate design*, understood as a discrete probability measure $\xi$ on $\mathscr {X}$, with finite support at *n* distinct points, known as support points.

$$\begin{aligned} \xi = \begin{Bmatrix} x_1&x_2&\cdots&x_n\\ w_1&w_2&\cdots&w_n \end{Bmatrix}\in \Xi , ~~~~ \sum _{i=1}^{n}w_i = 1, \end{aligned}$$

where $\xi (x_i)= w_i$ are the weights or proportions observed at each point $x_i$ and $\Xi$ is the set of all approximate designs in $\mathscr {X}$. Approximate designs are widely used in the OED literature due to their good properties for optimality calculation and verification. However, they must be adapted to the number of observations, *N*, to be carried out, which means rounding and fitting the weights. The calculation of efficient exact designs is considered in studies such as^21^. Therefore, in this paper, the designs are considered approximate.

In this study, the model considered is a non-linear regression model

$$\begin{aligned} y(x) =\eta (x, \theta )+ \varepsilon , ~~~~~~~\varepsilon \sim N(0, \sigma ^2), ~~~~ x\in \mathscr {X} \end{aligned}$$

where *y* is the response variable, *x* the vector of designable or controllable variables taking values in $\mathscr {X}$, $\eta$ the regression function and $\theta$ is the vector of unknown parameters of the model. It is assumed that the error, $\varepsilon$, follows a normal distribution with mean zero and constant variance $\sigma ^2$.

One of the main tools in this field is the Fisher Information Matrix (FIM)^22^. By definition, the inverse of the FIM is asymptotically proportional to the variance-covariance matrix of the estimators of the model parameters. Therefore, optimising the estimation of the parameters of a model is equivalent to optimising the inverse of the FIM. Assuming normality in the observations, the FIM can be defined as

$$\begin{aligned} M(\xi ) = \sum _{x\in \mathscr {X}}f(x)f^T(x)\xi (x), \end{aligned}$$

where $f(x)=(f_1(x),\ldots ,f_k(x))^T$ and each $f_i(x)=\left. \frac{\partial \eta (x,\theta )}{\partial \theta _i}\right| _{\theta ^{(0)}}$ corresponds to the partial derivative of the model with respect to the *i*-th parameter evaluated in $\theta ^{(0)}=(\theta _1^{(0)},\ldots ,\theta _k^{(0)})^T$ which are the initial values of the parameters, called nominal values. The need for these nominal values leads to obtaining locally optimal designs, since it depends on the choice of these values.

Different optimality criteria arise from the varied ways in which the FIM can be optimised. Each of these criteria is defined via a criterion function, $\Phi$, which is convex and lower bounded, and defined in $\mathscr {M}$, the set of all information matrices. The design $\xi ^\star$ which minimises $\Phi [M(\xi ^\star )]$ is called $\Phi$-optimal. This paper uses the most popular criterion in OED theory, the criterion of *D*-optimality. This is equivalent to minimising the volume of the confidence ellipsoid of the model parameter estimates, and so in practice it optimises the joint estimate of all the model parameters. Its criterion function is given by $\Phi _D[M(\xi )]=|M^{-1}(\xi )|$ when $|M(\xi )|\ne 0$ and 0 otherwise. Usually the determinant of the FIM is maximised, given the property that the determinant of the inverse of a matrix is the inverse of its determinant.

Furthermore, the criterion of *c*-optimality is also considered, which allows estimating a linear combination of the parameters $c^T\theta$ with minimum variance, where *c* is a known vector of constants. Its criterion function is given by $\Phi _c[M(\xi )]=c^TM^{-1}(\xi )c$.

By means of the General Equivalence Theorem (GET)^23,24^, it is possible to verify the $\Phi$-optimality of a design for those criteria based on the FIM, using the sensitivity function $\phi (x, \xi )$. A design is optimal when $\phi (x,\xi ^\star )\ge 0$ for all $x \in \mathscr {X}$ and $\phi (x,\xi ^\star )=0$ at the support points of $\xi ^\star$. In the case of the *D*-optimality criterion, the sensitivity function is expressed as $\phi (x, \xi ) = k-d(x, \xi )$, where $d(x, \xi ) = f^T(x)M^{-1}(\xi )f(x)$ is the generalised variance function, and *k* represents the number of parameters of the model. For the *c*-optimality criterion, it is defined as $\phi (x,\xi ) = c^TM^{-1}(\xi )c-(f^T(x)M^{-1}(\xi )c)^2$^25^.

The sensitivity function reflects the contribution of each design point to the variability in the estimation of the parameters, through the chosen optimality criterion. In the case of *D*-optimality, this is captured by the generalised variance function $d(x,\xi )$, which represents the variability associated with observing at point *x*. Therefore, it is desirable to include in the design those points where this variability is highest, i.e., where $d(x,\xi )$ is large and thus $\phi (x,\xi )$ is low. According to the General Equivalence Theorem, a design is optimal when $\phi (x,\xi ^\star ) \ge 0$ for all $x \in \mathscr {X}$ and $\phi (x,\xi ^\star ) = 0$ exactly at the support points of the optimal design. For the calculation of the optimal designs, it has been implemented in Python the Wynn-Fedorov algorithm, which is based on the GET^26,27^.

### Efficiency and sensitivity analysis

The $\Phi$-efficiency of a design $\xi \in \Xi$ is a measurement that evaluates the quality of the design $\xi$ as compared to the $\Phi$-optimal design, $\xi ^\star$. This efficiency takes values between 0 and 1, and is defined by the following expression:

3 $$\begin{aligned} \Phi \text {-Eff}(\xi |\xi ^\star ) = \frac{\Phi [M(\xi ^\star )]}{\Phi [M(\xi )]}. \end{aligned}$$

It is common to calculate the efficiency of a design with respect to the optimal design, although it is also possible to determine relative efficiencies between different designs. In the case of relative efficiencies, the set of possible values the efficiency can take is $[0, \infty )$.

The Atwood bound allows a minimum bound to be obtained for the efficiency of a design. It is used here to guarantee a minimum efficiency in cases where the Wynn-Fedorov algorithm has computational problems with convergence to the optimal design, as can occur in *c*-optimal designs, usually singular. This bound can be expressed as

$$\begin{aligned} \Phi \text {-Eff}(\xi |\xi ^\star ) \ge \frac{Tr[M(\xi )\nabla \Phi (\xi )]}{\max _{x\in \mathscr {X}} d(x,\xi )}, \end{aligned}$$

where *Tr* is the trace and $\nabla \Phi (\xi )$ is the gradient of the criterion function $\Phi$.

Given the uncertainty in the choice of nominal values for parameters, it is useful to perform a sensitivity analysis for the design with respect to these nominal values. This analysis studies the efficiency of the design when the chosen nominal value is not the true value of the parameter. This is done by varying the nominal value within a range considered of interest and calculating the efficiency of the design in each case. When the variations in efficiency are significant, it is said that the design is sensitive to uncertainty in that parameter, while in the opposite case it is said that the design is robust.

### Dataset

As a reference to explain a standard procedure followed in predictive microbiology for the estimation of the parameters of primary and secondary models, the experiment carried out by Tarlak et al.^16^ is taken as a case study.

This experiment first considers four isothermal experiments at temperatures of 4, 12, 20 and 28 °C (shown in Table 1). The results of these experiments give estimates for the kinetic parameters $y_0, y_{max}, \mu _{max}$ and $\lambda$ of the primary model expressed by Eq. (1) for each temperature, as shown in Table 2. Next, the estimate for the parameter $\mu _{max}$, associated with each temperature, is taken as the observed value, to obtain the estimates for the parameters of the secondary model ($b=0.0099\pm 4.95\times 10^{-4}$ and $T_{min}=-17.5\pm 1.7$ °C) defined by Eq. (2). Finally, these models are validated by expressing them in the form of differential equations using non-isothermal experiments.

Based on this procedure, different experimentation strategies addressed through OED are provided for each of the possible scenarios.

**Table 1:** Sample times of the isothermal experimental designs for each temperature in the experiment carried out by Tarlak et al.^16^.

T (°C)	Sampling points (h)							
4	0	24	60	96	144	192	240	
12	0	24	48	72	100	144	192	
20	0	12	24	36	48	72	96	120
28	0	12	24	36	48	60	72	84

**Table 2:** Estimation of the parameters for the Baranyi model in the isothermal experiment carried out by Tarlak et al.^16^.

T (°C)	$y_0$ (log CFU/g)	$y_{max}$ (log CFU/g)	$\mu _{max}$ (h^−1^)	$\lambda$ (h)
4	$7.08 \pm 0.06$	$8.65 \pm 0.05$	$0.043 \pm 0.007$	$44.1 \pm 10.4$
12	$7.08 \pm 0.06$	$10.14 \pm 0.05$	$0.091 \pm 0.006$	$33.2 \pm 3.6$
20	$7.06 \pm 0.12$	$10.71 \pm 0.13$	$0.134 \pm 0.015$	$23.5 \pm 4.7$
28	$7.05 \pm 0.07$	$10.64 \pm 0.07$	$0.202 \pm 0.013$	$20 \pm 1.9$

## Results

### Optimal designs in isothermal experiments

#### *D*-optimal designs

**Fig. 2:**
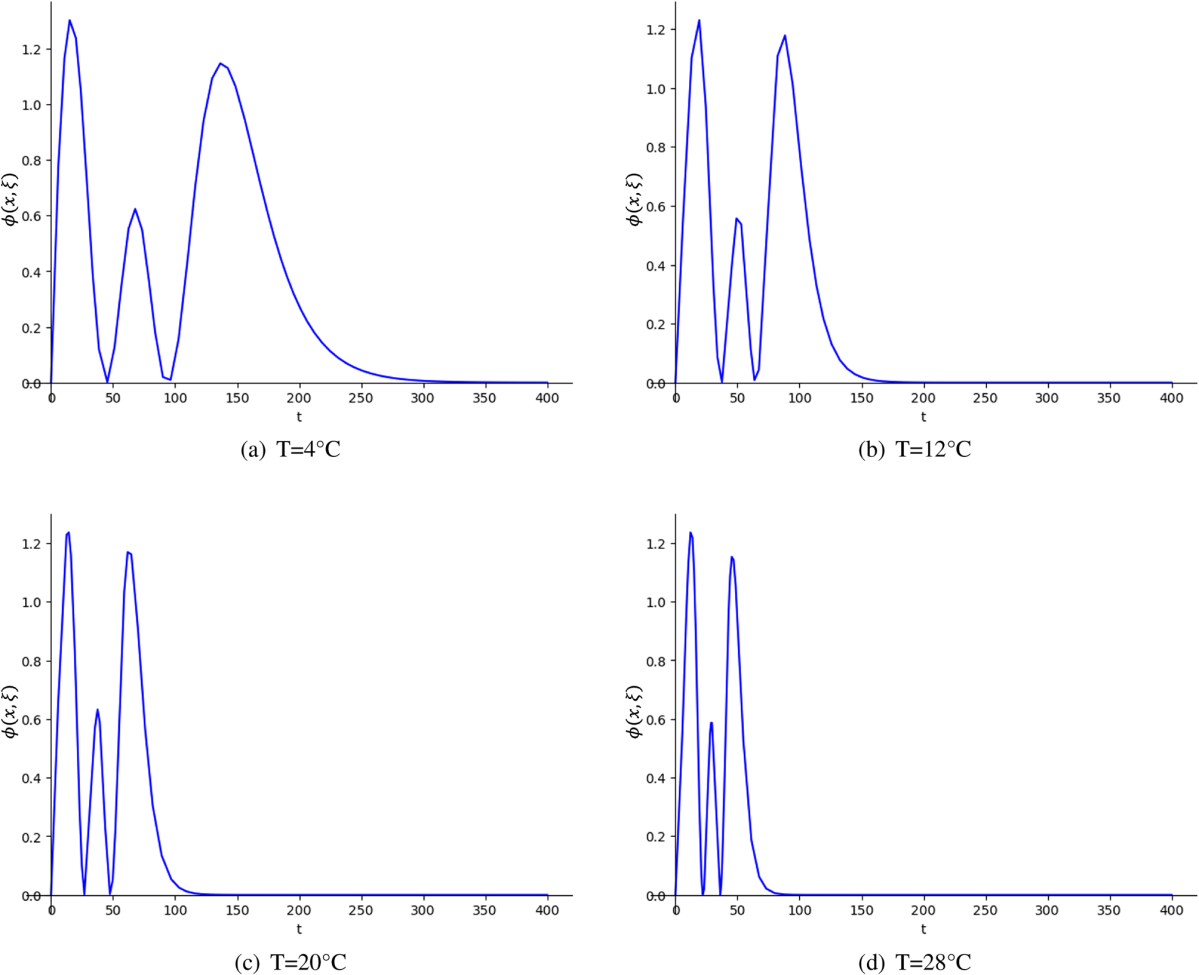
Sensitivity function $\phi (x,\xi )$ for *D*-optimal designs in Table 3.

Firstly, to estimate precisely all the model parameters ($y_0, y_{max}, \mu _{max}, \lambda$), *D*-optimal designs are given for each temperature proposed by Tarlak et al.^16^. The design space for time is considered from the beginning of the experiment (0 h) until the stationary phase of the model is reached, at which population growth no longer occurs. The parameter estimates provided by the authors for each temperature are considered as nominal values (Table 2). Table 3 shows the optimal designs obtained for each temperature, and the *D*-efficiency of the design proposed in the work of Tarlak et al.^16^ for each temperature. To calculate these efficiencies, their designs are considered approximate, with weight 1/*N* at each observation time. The *D*-optimal designs obtained are equally-weighted designs, with each support point corresponding to 25% of the total observations. These efficiencies indicate that there is a loss of efficiency between 14 and 20% compared to the obtained *D*-optimal designs. Figure 2a shows the sensitivity functions of the *D*-optimal designs. It is clear that the support points of the design are distributed similarly for the four cases, observing shorter distances between times the higher the temperature of the experiment. This behaviour is expected, since the higher the temperature, the faster the growth of microorganisms.

**Table 3:** Equally-weighted isothermal *D*-optimal designs for estimating $y_0$, $y_{max}$, $\mu _{max}$, and $\lambda$ for each temperature, and *D*-efficiencies of designs of Table 1 with respect to *D*-optimal designs.

T (°C)	*D*-optimal design	*D*-eff
4	$$\begin{Bmatrix} 0&45&92&485 \\ 1/4&1/4&1/4&1/4 \end{Bmatrix}$$	0.83
12	$$\begin{Bmatrix} 0&37.2&65.2&280 \\ 1/4&1/4&1/4&1/4 \end{Bmatrix}$$	0.82
20	$$\begin{Bmatrix} 0&27.5&48&185 \\ 1/4&1/4&1/4&1/4 \end{Bmatrix}$$	0.86
28	$$\begin{Bmatrix} 0&22.1&35.8&122 \\ 1/4&1/4&1/4&1/4 \end{Bmatrix}$$	0.8

#### *c*-optimal designs for $\mu _{max}$

Taking the $\mu _{max}$ parameter estimate as the observed value for estimating the secondary model parameters leads to ignoring its uncertainty in the second estimate. In this case, the best approach is to use an estimate of $\mu _{max}$ with minimum variance, and so *c*-optimality should be considered, which provides designs to estimate the parameter $\mu _{max}$ with minimum variance for each temperature. Table 4 shows these *c*-optimal designs for each temperature. It should be noted that in this case, equally-weighted designs are no longer obtained, but rather certain support points have a greater weight than others in the design. Thus, the central points of the design account for between 65 and 70% of the observations to be made. This is related to the physical meaning of the kinetic parameter $\mu _{max}$, as it defines the slope of the growth curve in the central phase, the exponential phase. A certain similarity can also be seen between the points and the weights of the various designs, and the phenomenon already mentioned for the *D*-optimal designs, whereby the higher the temperature the shorter the time between support points, is seen again. These designs allow the parameters of the secondary model to be estimated based on the values of $\mu _{max}$ with minimal uncertainty. The third column of Table 4 gives the *c*-efficiency of the designs proposed by the authors (Table 1) with respect to the *c*-optimal design; that is, the goodness measure of how well they estimate the parameter $\mu _{max}$ with respect to the *c*-optimal design. These *c*-efficiencies, below 50% in all cases, indicate that the parameter estimation of these designs entails 50% more variability than that obtained with the *c*-optimal design.

It is clear that these designs do not provide accurate individual estimations of the parameter $\mu _{max}$. The last column of Table 4 shows the *c*-efficiency of the *D*-optimal designs obtained in Section “*D*-optimal designs”, confirming that, even using these designs, the estimation of parameter $\mu _{max}$ is considerably more accurate than with those previously mentioned.

**Table 4:** c-optimal designs for estimating $\mu _{max}$ and *c*-efficiencies of designs of Table 1 and *D*-optimal designs of Table 3 with respect to *c*-optimal designs.

T (°C)	*c*-optimal design	Tarlak *c*-eff	*D*-optimal *c*-eff
4	$$\begin{Bmatrix} 0&41&97.4&300 \\ 0.14&0.295&0.36&0.205 \end{Bmatrix}$$	0.47	0.82
12	$$\begin{Bmatrix} 0&34.6&67.34&200 \\ 0.145&0.33&0.345&0.165 \end{Bmatrix}$$	0.44	0.82
20	$$\begin{Bmatrix} 0&25.8&49.5&125 \\ 0.137&0.34&0.36&0.153 \end{Bmatrix}$$	0.44	0.8
28	$$\begin{Bmatrix} 0&21.15&36.97&100 \\ 0.145&0.337&0.342&0.151 \end{Bmatrix}$$	0.42	0.81

### Optimal designs controlling temperature and time

In the optimal designs calculated in the previous section, the temperatures considered are the ones fixed by the experimenters in the work of Tarlak et al.^16^. It is possible, however, that the temperatures chosen in the experiments also influence the estimation of the model parameters. Therefore, to accurately estimate the parameters of the primary model (function of time) and the secondary model (function of temperature), the most suitable solution is to design over both variables, time and temperature. To do this, the design is calculated for the primary model (Eq. (1)) in which the parameter $\mu _{max}$ is substituted for the secondary model (Eq. (2)). Calculating the *D*-optimal design for this extended model provides the design that allows accurate estimation of the parameters $y_0$, $y_{max}$, $\lambda$, *b* and $T_{min}$, jointly. In this way, not only the times but also the temperatures at which the experiment should be carried out are obtained, thus eliminating the constraint of having to experiment at the fixed temperatures of 4, 12, 20 and 28 °C. The design space considered for temperature is [4 °C, 28 °C].

#### *D*-optimal design

In order to calculate the *D*-optimal isothermal design in temperature and time, we take as nominal values of the primary model parameters the mean of the estimates provided by Tarlak et al.^16^. For the parameters of the secondary model, *b* and $T_{min}$, the nominal values taken are the estimates provided in that work. These nominal values are provided in Table 5.

**Table 5:** Nominal values for the parameters in the Baranyi primary model and Ratkowsky secondary model, for *D*- and *c*-optimal desings controlling temperature and time.

Parameter	$y_0$ (log CFU/g)	$y_{max}$ (log CFU/g)	$\lambda$ (h)	*b*	*T*_*min*_ (°C)
Nominal value	7.07	10.03	30.2	0.0099	$-17.5$

**Fig. 3:**
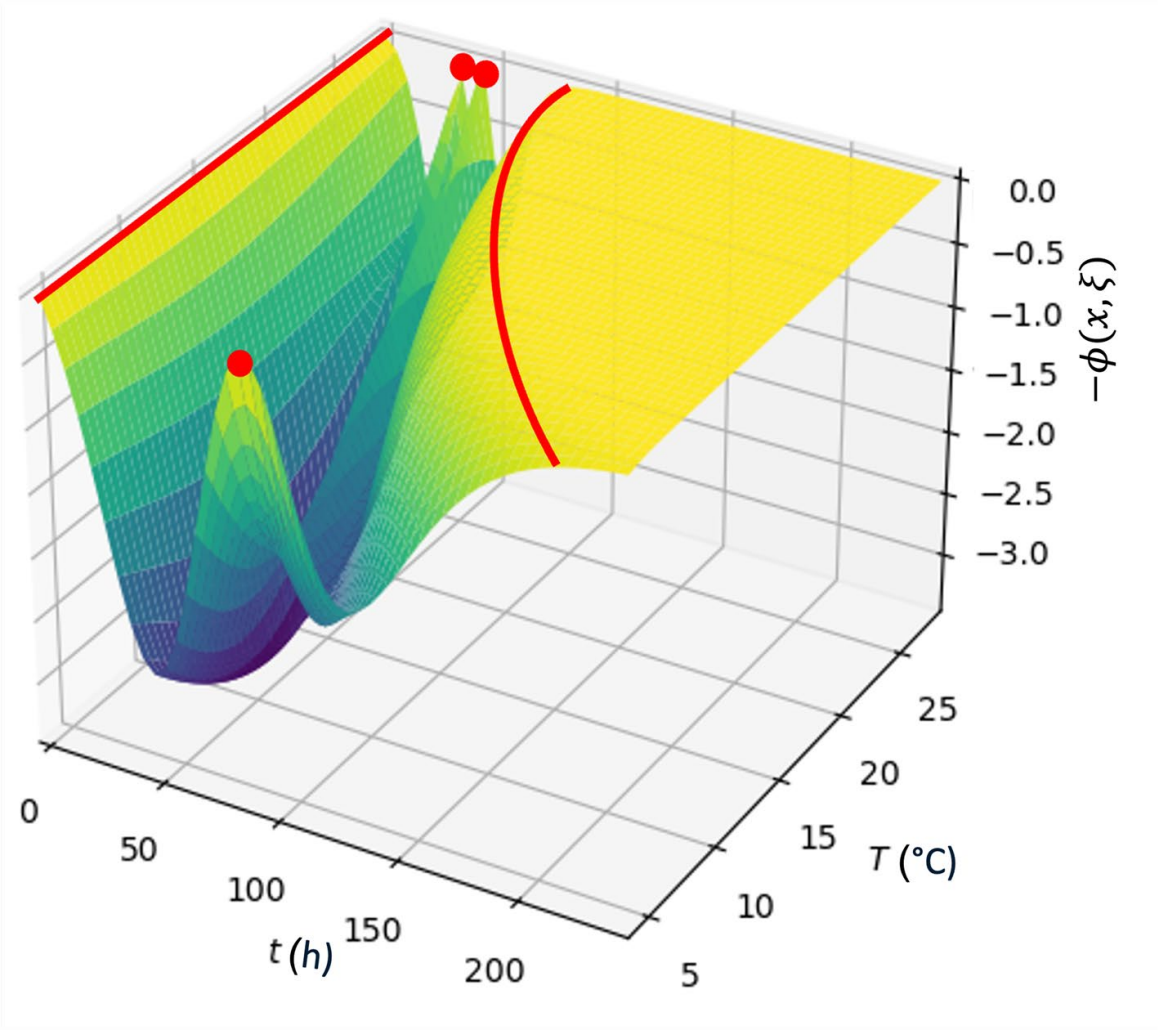
Opposite of the sensitivity function, $-\phi (x,\xi )$, of the isothermal *D*-optimal design in temperature (*T*) and time (*t*), $\xi ^\star _T$. Red marks stand for the optimal design support points.

Figure 3 shows, for ease of visualisation, the opposite of the *D*-optimal design sensitivity function where the maxima, marked in red, correspond to the support points of the design. This design leaves the temperature of the first point to the experimenter’s choice, since the maximum is reached for time 0 at any temperature. In the same way, the last support point of the design could be freely chosen from those that make up the flat surface that extends to the end of the experimentation time, that is, the time until which it would be necessary to observe in order to make the final observation. Considering that the optimal cost option is to stop experimenting as soon as possible, the optimal lengths of the experiments are calculated for the full temperature range (4–28 °C) for which optimality is achieved in the design. The relationship between the temperature at which the experiment is to be performed and the final experimental time is then adjusted, using these values, via the function $t_f(T)=744.7-186.8\ln (T)$. This relationship also explains the final experimental time of the experiment for the isothermal *D*-optimal designs in Table 3. Thus, the *D*-optimal design obtained is

$$\begin{aligned} \xi ^\star _T = \begin{Bmatrix} (T,0)&(4,87.88)&(28,31.17)&(28,43.75)&(T,t_f(T)) \\ 0.2&0.2&0.2&0.2&0.2 \end{Bmatrix}. \end{aligned}$$

Note that this design could be applied when performing only two isothermal experiments, one at 4 °C and the other at 28 °C. Since at 0 h it would be possible to experiment at any temperature, either 4 °C, or 28 °C could be chosen. For the final experimental point, if the temperature is chosen to be 4 °C, the final experimental time would be $t_f(4)=485.74$ h, while if the decision is to experiment at 28 °C, it would be $t_f(28)=122.24$ h. Thus, for example, if the cost of increasing the temperature were high, the choice could be made to carry out three independent observations at 4 °C at 0 h, 87.88 h and $t_f(4)=485.74$ h and two independent observations at 28 °C, at 31.17 h and 43.75 h. While if the time cost is higher, then observations could be made at 4 °C at two time points, 0 h and 87.88 h, and at 28 °C at three points 31.17 h, 43.75 h and $t_f(28)=122.24$ h. Any of the above combinations maintains the optimality of the design, that is, it optimises precision in the joint estimation of all parameters.

For the purposes of making a comparison, the Tarlak design in two variables is considered to be the union of the four designs that they propose at each of the temperatures (Table 1). Thus, each experimental point has an associated weight of 1/30. The *D*-efficiency of this design with respect to $\xi ^\star _T$ is 0.58. In the case of the joint *D*-optimal design, from the union of the four designs described in Table 3, its *D*-efficiency with respect to $\xi ^\star _T$ is 0.65. The efficiencies indicate that the designs applied previously, with the imposition of the fixed temperatures, are rather limited when it comes to accurately estimating the parameters of both models together.

#### Sensitivity analysis

**Fig. 4:**
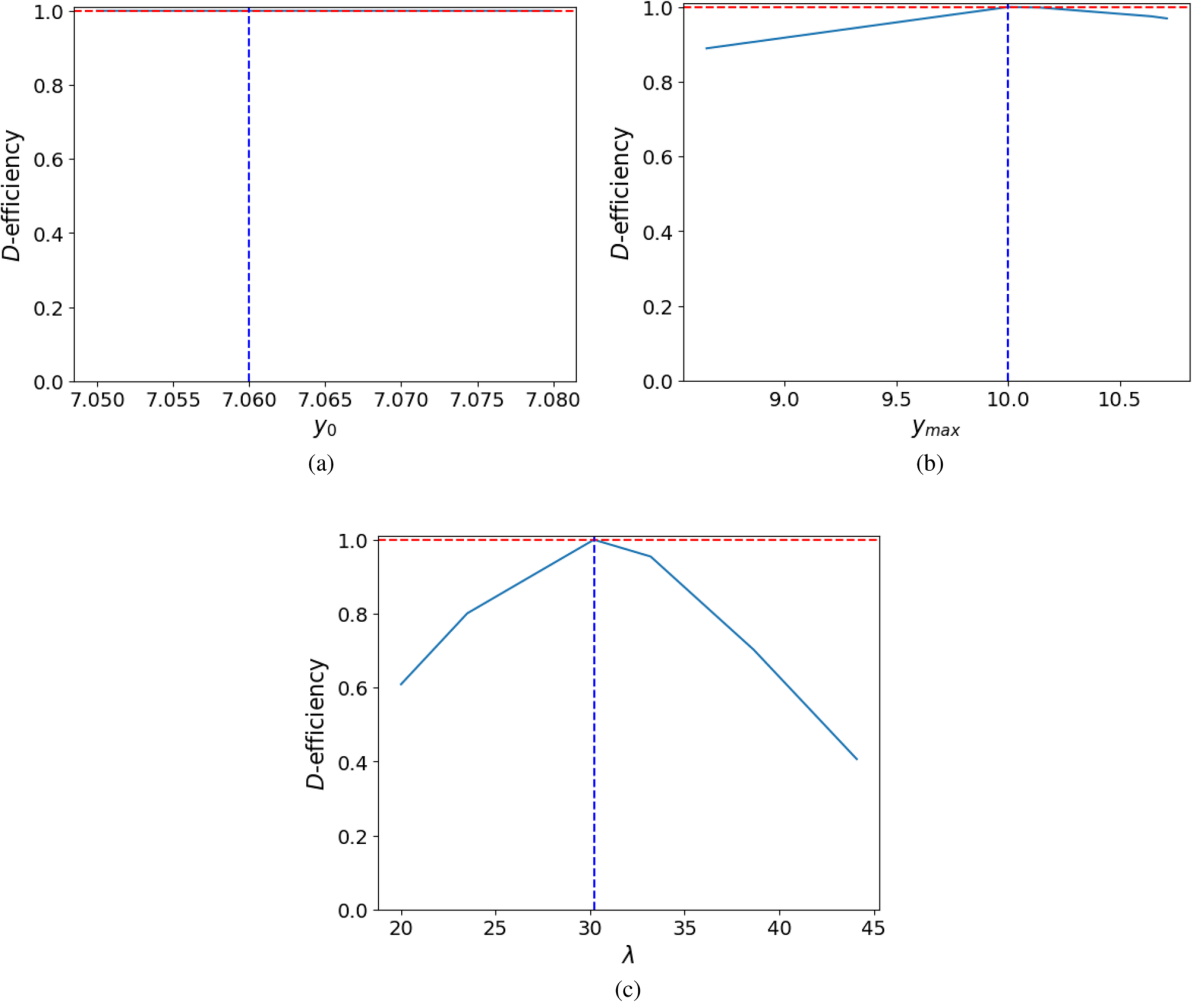
*D*-efficiencies for the sensitivity analysis of $\xi ^\star _T$ with respect to the nominal values of the primary model parameters $y_0$ (**a**), $y_{max}$ (**b**) and $\lambda$ (**c**).

Since the nominal values of the primary model parameters, $y_0$, $y_{max}$ and $\lambda$, have been taken as the mean of the estimates of these parameters obtained by Tarlak et al.^16^ (Table 2), it is of interest to study the sensitivity of the *D*-optimal design $\xi ^\star _T$ to the range of values of the estimates of these parameters. To carry out this analysis, the efficiency of the obtained *D*-optimal design, $\xi ^\star _T$, is calculated with respect to the *D*-optimal design for another nominal value of this parameter using Eq. (3). Figure 4 provides a graphical representation of the sensitivity analysis. The red line indicates the maximum achievable efficiency, which is equal to 1. For this reason, the optimal design achieves maximum efficiency for the initial nominal value (marked by the vertical blue dashed line). As shown in Fig. 4, this design undergoes a greater loss of efficiency for variations in the nominal value of the parameter $\lambda$, and is more pronounced in the case of underestimation of this parameter, while it behaves robustly for the range of estimates of the parameters $y_0$ and $y_{max}$. As a solution to the loss of efficiency with respect to the uncertainty in the nominal values, Muñoz del Río et al.^28^ describe a methodology through which the optimal design is made robust.

#### Analysis of the relationship between temperature and final experimental time

**Fig. 5:**
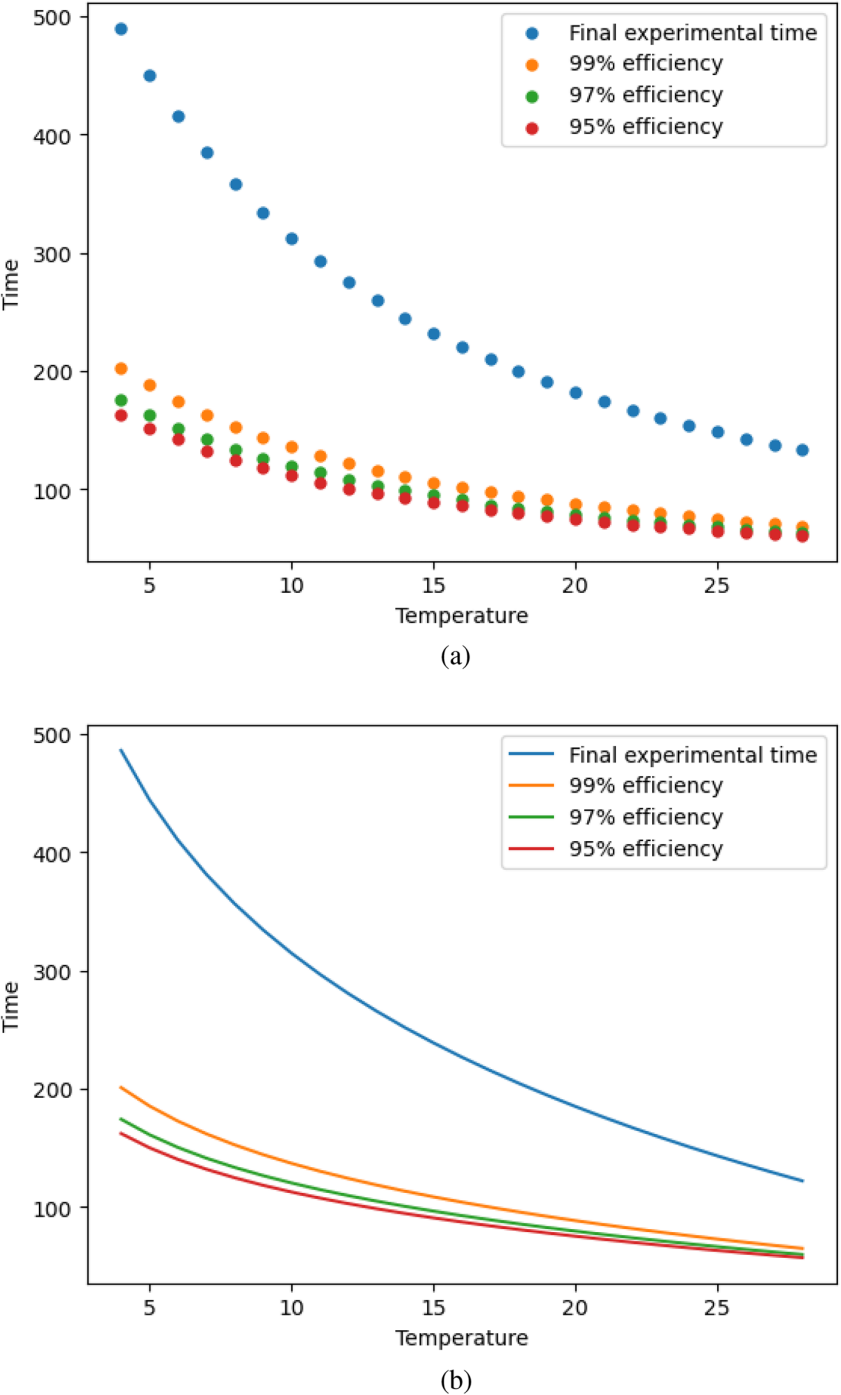
Final time-saving curves of the experiment ensuring minimum efficiency. (**a**) Real temperature-time values obtained for each minimum guaranteed efficiency. (**b**) Mathematical function of final experimental time with respect to temperature expressed in Table 6.

As shown in Section “*D*-optimal design”, $\xi ^\star _T$ has a support point where the experiment time depends on the temperature at which the experiment is being carried out. As shown in the results, as the temperature increases, the final experimental time decreases. Furthermore, this relationship between final experimental time and temperature has been adjusted using a logarithmic model. It seems logical that for cost-saving reasons the experimenter would therefore seek to shorten the experiment as much as possible. Therefore, based on the $\xi ^\star _T$ design, an efficiency analysis was carried out to determine the final experimental times that lead to designs with high efficiency, and so can lead to greater time savings. Figure 5a shows the behaviour of the relationship between temperature and final experimental time for both the $\xi ^\star _T$ design and for designs with 99%, 97% and 95% efficiency. It can be seen that assuming a 1% efficiency loss already significantly reduces the final experimental times with a time saving of between 48 and 58%. Table 6 also shows the adjustments to these relationships (represented in Fig. 5b) along with the RMSE value and the relative savings in experiment time. It should be noted that the best adjustments found after considering different options, such as polynomial and exponential functions, are shown.

**Table 6:** Relationship between temperature and final experimental time based on efficiency.

Minimum efficiency (%)	$t_f(T)$	RMSE	Minimum saving (%)	Maximum saving (%)
99	$297.8-69.8\ln (T)$	1.55	48.5	58.6
97	$255.7-58.72\ln (T)$	1.62	52.9	64
95	$236.9-53.88\ln (T)$	2.01	54.5	66.7

First column: minimum guaranteed efficiency. Second column: mathematical expressions of final experimental time as a function of temperature. Third column: Root Mean Square Error (RMSE) associated with each function. Fourth and fifth column: minimum and maximum relative time saving with respect to the optimal experimental design $\xi ^\star _T$.

#### *c*-optimal design for *b* and $T_{min}$

Although the solution proposed in Section “*c*-optimal designs for $\mu _{max}$” represents a significant improvement when adjusting the parameters of the secondary model, a design solely intended to estimate these parameters solves the problem of avoiding the uncertainty associated with the estimation of the parameter $\mu _{max}$ when it is taken as an observed value. The *c*-optimal design, obtained using the nominal values referenced in Table 5, of both parameters simultaneously is proposed for this purpose

$$\begin{aligned} \xi ^\star _{b,T_{min}} = \begin{Bmatrix} (T,0)&(4,111.5)&(26.3,60.7)&(28, 44.4)&(28,160) \\ 0.188&0.287&0.32&0.196&0.0038 \end{Bmatrix}. \end{aligned}$$

The *c*-efficiency of the *D*-optimal design, $\xi ^\star _T$, with respect to $\xi ^\star _{b,T_{min}}$ is 0.015, indicating very poor precision in estimating the secondary model parameters with the design intended for accurate estimation of all parameters together. The option of calculating the *c*-optimal for *b* and $T_{min}$ separately was also explored. In the latter case, the design is the same as the joint *c*-optimal design for *b* and $T_{min}$. In the case of the *c*-optimal design for estimating parameter *b*, its *c*-efficiency with respect to the *c*-optimal design for both parameters, $\xi ^\star _{b,T_{min}}$, is 0.41, so it does not represent a direct solution to the problem.

## Discussion

This study addresses the experimental procedure used in predictive microbiology for estimating model parameters from the OED perspective. The Baranyi model is considered as the primary model, and the Ratkowsky square-root model as the secondary model applied to the case study set out in^16^. Firstly, considering the isothermal experiments with the temperatures fixed and the objective being to estimate all the parameters of the primary model, the *D*-optimal design is provided for each temperature. The *D*-efficiency (Table 3) of the designs used in practice to estimate these parameters shows a loss of efficiency of around 20% compared to the optimal ones, which results in lower precision in the joint estimation of the parameters. This loss in *D*-efficiency results, in practical terms, in a larger volume of the confidence ellipsoid for the model parameter estimators. Consequently, Tarlak’s designs imply wider confidence intervals, less precise predictions than optimal designs. On the other side, if the optimal designs are used approximately 20% fewer experimental replicates are needed to achieve the same estimation accuracy, leading to a significant reduction in experimental costs. In predictive microbiology, the estimation of the parameters of the secondary model is carried out by fitting the values obtained from the estimation of the parameter $\mu _{max}$ in the previous step for each temperature. This fitting assumes the estimates of a parameter to be observed values, which is associated with an estimation error that is not considered. This paper proposes the use of the *c*-optimal design to obtain an estimate with minimum variance of the parameter $\mu _{max}$. In this way the fitting is made on a value with lower associated uncertainty. The results of the *c*-efficiency (Table 4) of these designs compared to those used in practice show a reduction of more than 50% in the number of required observations.

Now, in order to provide fair comparisons, the temperatures at which these experiments are designed are those taken from the study with which the results are compared. However, OED allows for the precise estimation of all parameters in a direct manner by designing in two variables, that is, providing not only the times but also the temperatures at which the experiment should be carried out. If the kinetic parameter ($\mu _{max}$) is replaced in the primary model by the secondary model, it provides a extended model in two variables, time and temperature, with all the parameters of interest. The *D*-optimal design in two variables, $\xi ^\star _T$, results in a 42% reduction in the number of observations with respect to the designs used in practice. These reductions not only improve the statistical quality of the parameter estimates but also lead to significant savings in time, cost, and laboratory resources, making the experimental process more efficient and feasible, especially in industrial or regulatory settings. Since this optimal design depends on the choice of initial values for the parameters, a sensitivity analysis of the design is carried out against uncertainty in these values. This analysis shows that the design is robust for all parameters except $\lambda$. To make the design more robust, the methodology developed by Muñoz del Río et al.^28^ could be applied, in which points are added to the support of the optimal design to minimise its loss of efficiency with respect to the uncertainty in the selected parameter(s). This methodology is based on the idea of the maximin criterion with a previous step, which guarantees computational savings, in which the candidate points to enter the design are preselected.

Two-variable *D*-optimal design leaves the experimenter to choose two of the observation points, the initial and final points. For the initial point (0 h), the experimenter can choose the temperature at which the sample is taken. For the final point, due to the construction of the design, it can be observed that there is a relationship between the temperature at which the experiment is carried out and its final experimental time. In order to save costs in the experiments, different reductions in the final experimental time of the experiment are proposed (Fig. 5), ensuring a minimum efficiency of the resulting design. These reductions show a minimum saving of 48% in time with respect to the optimal time, with minimal losses in efficiency (between 1 and 5%). In addition, these relationships between temperature and final experimental time are expressed mathematically for various efficiencies (Table 6). This is of interest because the experimenter, knowing the relationship of costs of the experiment relative to time or temperature, can choose between waiting longer and experimenting at a lower temperature, or shortening the time of the experiment by increasing the temperature of the final observation.

Finally, in the case where the goal of the study is only to estimate the parameters of the secondary model, the *c*-optimal design of both parameters in two variables is proposed, $\xi ^\star _{b,T_{min}}$. The *c*-efficiency of the previous *D*-optimal design, $\xi ^\star _T$, with respect to this one is 1.5%, that is, when the only concern is to estimate these two parameters, *D*-optimal design is not very efficient and a design intended solely for this estimation is necessary.

The methodology presented in this work focuses on the construction of approximate optimal designs, which provide the values of the controllable variables where observations should be taken, along with the proportion of replicates at each value. In practical terms, the efficiency of a design reflects the quality of parameter estimation when the total number of observations is fixed. Therefore, it quantifies the gain in estimation accuracy achieved by observing at certain points versus others. For example, if a design has 50% efficiency relative to the optimal design, this means that the optimal design can achieve the same level of estimation precision using only half the number of observations. This translates directly into savings in experimental effort and cost. However, in cases where experiments have already been conducted, it is still possible to apply this framework to evaluate the quality of the existing design. When the optimal design is known, comparing it with the current design allows one to quantify potential gains in efficiency and estimate how many observations could have been saved without compromising precision. Additionally, when the optimal design is not available, tools such as the Atwood’s bound can provide a lower bound on the efficiency of a given design. Moreover, methodologies such as sequential or adaptive design can be employed starting from existing designs to identify new observation points that incrementally guide the experiment towards optimality. These approaches offer a practical path for improving parameter estimation even after initial experimentation has been performed.

As future work, it would be of great interest to carry out experiments based on the proposed optimal designs and estimate the model parameters using the resulting data. This would allow an empirical evaluation of the expected improvements in parameter estimation precision, for example by comparing the width of the confidence intervals with those obtained using non-optimal designs.

## Data Availability

The data supporting the findings of this study are derived from previously published research. Specifically, the data were obtained from the study by Tarlak et al., published in Food Research International, and are available at the following link: https://www.sciencedirect.com/science/article/abs/pii/S0963996919307987 with DOI https://doi.org/10.1016/j.foodres.2019.108912. Additionally, the relevant data are provided within our manuscript.
